# Heme Oxygenase-1 and Its Role in Colorectal Cancer

**DOI:** 10.3390/antiox12111989

**Published:** 2023-11-10

**Authors:** Jörg Fahrer, Simon Wittmann, Ann-Cathrin Wolf, Tina Kostka

**Affiliations:** Division of Food Chemistry and Toxicology, Department of Chemistry, RPTU Kaiserslautern-Landau, Erwin-Schrödinger Strasse 52, D-67663 Kaiserslautern, Germany; wittmann@chemie.uni-kl.de (S.W.); acwolf@rptu.de (A.-C.W.)

**Keywords:** heme oxygenase-1, heme, colorectal carcinogenesis, reactive oxygen species, DNA damage, inflammation, ferroptosis, epithelial-mesenchymal transition, carbon monoxide

## Abstract

Heme oxygenase-1 (HO-1) is an enzyme located at the endoplasmic reticulum, which is responsible for the degradation of cellular heme into ferrous iron, carbon monoxide and biliverdin-IXa. In addition to this main function, the enzyme is involved in many other homeostatic, toxic and cancer-related mechanisms. In this review, we first summarize the importance of HO-1 in physiology and pathophysiology with a focus on the digestive system. We then detail its structure and function, followed by a section on the regulatory mechanisms that control HO-1 expression and activity. Moreover, HO-2 as important further HO isoform is discussed, highlighting the similarities and differences with regard to HO-1. Subsequently, we describe the direct and indirect cytoprotective functions of HO-1 and its breakdown products carbon monoxide and biliverdin-IXa, but also highlight possible pro-inflammatory effects. Finally, we address the role of HO-1 in cancer with a particular focus on colorectal cancer. Here, relevant pathways and mechanisms are presented, through which HO-1 impacts tumor induction and tumor progression. These include oxidative stress and DNA damage, ferroptosis, cell cycle progression and apoptosis as well as migration, proliferation, and epithelial-mesenchymal transition.

## 1. Introduction to HO-1 and Its Role in Physiology and Pathophysiology

Heme oxygenase (HO) is a metabolic enzyme and central player in the mammalian stress response, which was first described by Tenhunen and colleagues in 1968 [1,2]. The two major isoforms are HO-1 and HO-2, which are encoded by different genes (*HMOX1* and *HMOX2*, respectively). Both isoforms catalyze the rate-limiting degradation of intracellular heme to carbon monoxide (CO), ferrous iron (Fe^2+^) and Biliverdin-IXα (BV) [3,4]. While HO-1 is strongly inducible by its substrate heme and various (stress) stimuli including cadmium, hydrogen peroxide, hypoxia and inflammation, HO-2 is constitutively expressed and its level remains unchanged after cellular stress [5,6]. Due to a conserved carboxyl-terminal membrane-binding motif, HO-1 and HO-2 both localize to the endoplasmic reticulum (ER) [7,8,9]. However, a stress-induced redistribution of HO-1 to mitochondria and the nucleus was shown in several studies [8,9,10,11,12]. For instance, hypoxia or heme can trigger the nuclear translocation of HO-1 associated with a reduction of its activity [9]. The expression and activity of HO-1 depends on the cellular microenvironment, cell type, duration and intensity of stimuli exposure and can rise up to 100-fold [5,6]. HO-1 expression is regulated by upstream protein kinases as well as transcription factors [5] (see Section 3). 

HO-1 is found in most human tissues, with the highest activity in the spleen, liver, and bone marrow [13]. The importance of HO-1 in human physiology was first demonstrated by a rare case of HO-1 deficiency in a six-year-old boy who suffered from symptoms including anemia, endothelial cell damage, and iron deposition [14]. In addition, knockout mouse experiments showed that homozygous *Hmox1*^−/−^ offspring of heterozygously mated *Hmox1*^+/−^ mice had reduced survival, whereas the mating of *Hmox1*^−/−^ animals did not produce any living offspring at all [15,16]. Adult *Hmox1*-deficient animals were thinner, less active, reproduced poorly, and died earlier than *Hmox1*^+/−^ and *Hmox1*^+/+^ animals. Homozygous *Hmox1*^−/−^ mice suffered from accumulation of non-heme iron in the renal proximal cortical tubules and liver, as well as hypoferremia and anemia, indicating an important role of HO-1 in iron homeostasis. Furthermore, HO-1 deficient mice were prone to liver damage and mortality upon induction of oxidative stress [15,16].

As shown in a sepsis rat model, HO-1 expression was higher in the duodenum, jejunum and ileum than in the colon after lipopolysaccharide (LPS)-treatment. LPS-induced mucosal injury and inflammation was increased in the colon, suggesting a protective effect of HO-1 in the upper intestine [17]. HO-1 is involved in maintaining the cellular barrier function, in chronic inflammatory diseases as well as bacterial defense mechanisms by macrophages [18,19,20] (details given in Section 6). Seiwert and colleagues further elucidated the protective role of HO-1 against oxidative stress triggered by hemin in human colonic epithelial cells. Chemical inhibition of HO-1 by zinc protoporphyrine IX (ZnPP) or its genetic knockdown strongly increased reactive oxygen species (ROS) levels, DNA damage and cytotoxicity, demonstrating the pivotal role of HO-1 in cytoprotection against toxic heme in colonocytes [21]. HO-1 also plays an important role in bone marrow stem cell differentiation [13]. HO-1 induction in human bone marrow-derived mesenchymal stem cells (MSCs) was observed to enhance their differentiation into osteoblasts, whereas inhibition of HO-1 resulted in increased adipocyte formation [22]. The upregulation of HO-1 and activity decreased adipocyte differentiation with an associated increase in small healthy lipid droplets. Furthermore, HO-1 induced reductions in tumor necrosis factor-α (TNFα) levels and adipocyte hypertrophy, whereas adiponectin levels and expression of key genes of the canonical WNT pathway were increased [22]. Moreover, HO-1 was described to influence the bone metabolism and inhibit osteoclastogenesis via preventing differentiation of osteoclast precursors to osteoclasts in murine spleen cells [23]. Taken together, HO-1 is involved in several physiological processes, by acting as a protective factor against ROS formation and inflammation. 

## 2. Structure and Function of HO-1

**Figure 1:**
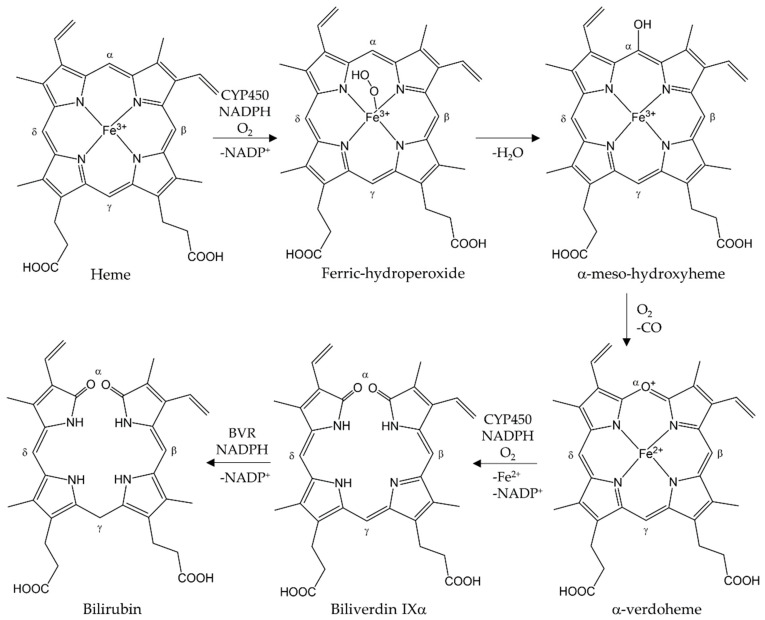
Degradation of heme catalyzed by HOs. The final products are CO, Fe^2+^ and biliverdin IXα, which is reduced to bilirubin by biliverdin reductase (BVR). Created with BioRender.com, accessed on 26 October 2023.

*HMOX1* is located on chromosome 22 (22q12) in the human genome [24] and encodes for the HO-1 protein with a size of 32 kDa. HO-1 belongs to the class of heme-hydrogen donor-oxygen oxidoreductases (EC 1.14.99.3) and catalyzes the degradation of heme in its oxidized form, i.e., hemin (Fe^3+^ protoporphyrine) [25]. HO-1 is expressed on the surface of the ER and its catalytic center is oriented towards the cytosol [26]. The single reaction steps catalyzed by HO-1 are illustrated in the figure below (Figure 1). 

HO-1 forms a complex with hemin, and ferrous iron is formed through the first electron donation from NADPH by NADPH cytochrome P450 reductase. Molecular oxygen binds to the complex, yielding a metastable oxy-form, after which the iron-bound oxygen is converted to a hydroperoxide intermediate (Fe^3+^-OOH) through further electron transfer from NADPH cytochrome P450 reductase and a proton from a surrounding water molecule [27]. Terminal oxygen of Fe^3+^-OOH attacks the α-meso carbon of the porphyrin ring, thereby producing ferric α-meso-hydroxyheme. Subsequently, a ferrous verdoheme-HO complex and CO are formed through the reaction with molecular oxygen. The oxy-verdoheme-HO complex is converted into a ferric iron-biliverdin chelate through a hydroperoxide intermediate that is still bound to HO. The iron in ferric biliverdin is reduced to the ferrous state by the reductase, releasing Fe^2+^ and BV. Using in vitro experiments, the release of BV from the enzyme was revealed as the rate-determining step [27,28]. BV is then reduced to bilirubin (BR) by biliverdin reductase (BVR), which is glucuronidated in the liver and finally excreted via the bile.

## 3. Regulation of HO-1 Expression

### 3.1. Transcriptional Regulation of HMOX1

The expression of *HMOX1* is mainly regulated on the transcriptional level. Owing to different binding sites in the 5’-flanking region of the promoter, expression of *HMOX1* can be regulated by several transcription factors such as nuclear factor erythroid 2-related factor 2 (Nrf2), activator protein-1 (AP-1), and nuclear factor-κB (NF-κB), which bind to specific sequences in the enhancer elements E1 and E2 [29,30].

**Figure 2:**
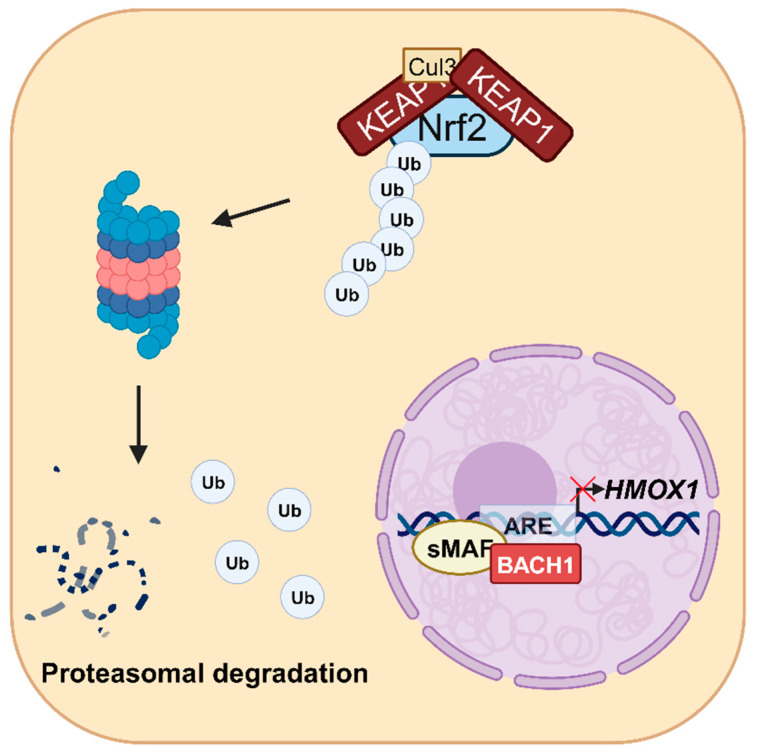
KEAP1/Nrf2-pathway under homeostatic conditions and *HMOX1*. Under normal conditions, Nrf2 in the cytosol is tagged with ubiquitin units by a complex consisting of Kelch-like ECH-associated protein 1 (KEAP1) and the ubiquitin E3 ligase Cullin 3 (Cul3). Cul3-catalyzed polyubiquitination of Nrf2 leads to its proteasomal degradation and prevents its nuclear translocation. In the nucleus, Nrf2-mediated *HMOX1* transcription is repressed (indicated by the red cross) by a heterodimer of small musculoaponeurotic fibrosarcoma protein (sMaf) and BTB and CNC homology 1 (BACH1) that binds to two enhancer sequences of the antioxidant response element (ARE). Created with BioRender.com, accessed on 25 October 2023.

The cap’n’collar (CNC) subfamily of basic-region leucine zipper (bZIP) transcription factors Nrf2 and BACH1 (BTB and CNC homology 1) are the most important transcriptional regulators of *HMOX1* [31,32,33]. Under normal conditions, redox-sensitive Nrf2 resides in the cytosol, where it is marked by Kelch-like ECH-associated protein 1 (KEAP1) for polyubiquitination catalyzed by the ubiquitin E3 ligase cullin 3 (Cul3), leading to its subsequent proteasomal degradation [34] (Figure 2). 

**Figure 3:**
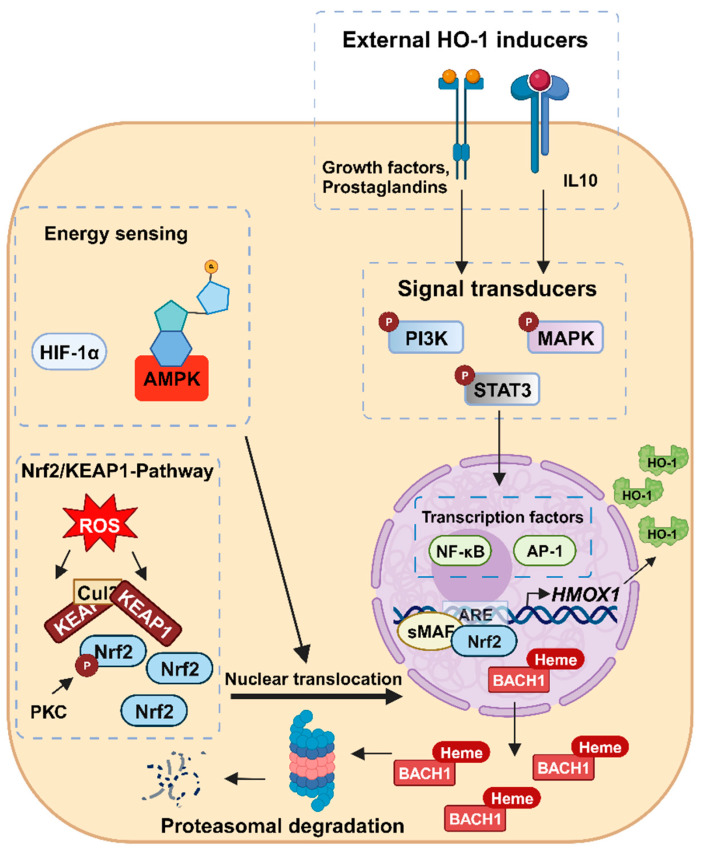
Multiple layers of *HMOX1* transcriptional regulation under cellular stress. Reactive oxygen species (ROS) react with cysteine residues of KEAP1, leading to the dissociation of the Kelch-like ECH-associated protein 1 (KEAP1)-ubiquitin E3 ligase cullin 3 (Cul3) complex from Nrf2. In response to oxidative stress, protein kinase C (PKC) is able to phosphorylate Nrf2, which also promotes its dissociation from KEAP1. Stabilized Nrf2 translocates into the nucleus, where it forms a heterodimer with small musculoaponeurotic fibrosarcoma protein (sMaf) proteins. This heterodimer binds to the enhancer region of the antioxidant response element (ARE) and induces *HMOX1* transcription. Heme can bind to the negative regulator BTB and CNC homology 1 (BACH1), leading to its dissociation from the BACH1-sMAF dimer, followed by its nuclear export and proteasomal degradation. The transcription factor hypoxia-inducible factor-1 (HIF-1α) and the enzyme AMP-activated protein kinase (AMPK) were shown to activate *HMOX1* expression in an Nrf2-dependent manner. In addition, extracellular stimuli such as interleukin-10 (IL-10) can induce *HMOX1* transcription via mitogen-activated protein kinases (MAPKs), signal transducer and activator of transcription (STAT)-3 and phosphatidylinositol 3-kinase (PI3K) pathways. Transcription factors such as activator protein-1 (AP-1) and nuclear factor-κB (NF-κB) can bind directly to specific sequences in the enhancer element to promote *HMOX1* gene expression. Created with BioRender.com, accessed on 25 October 2023.

In the presence of oxidative or electrophilic reactants as well as other Nrf2 activators, the cysteine residues of KEAP1 are modified, resulting in loss of ubiquitin E3 ligase activity of the KEAP1-CUL3 complex and stabilization of Nrf2 [29,31] (Figure 3). The stabilized Nrf2 then translocates into the nucleus, where it forms a heterodimer with small musculoaponeurotic fibrosarcoma (sMaf) proteins. The Nrf2-sMaf dimer induces transcription of several antioxidant genes such as *HMOX1* by binding to the antioxidant response element (ARE) [31,35,36]. Normally, a heterodimer consisting of BACH1 and one of the three sMaf proteins MafF, MafG, or MafK binds to the ARE in two enhancer regions of *HMOX1* and represses its transcription [29,31]. However, heme can cause dissociation of the heterodimer by direct binding to BACH1. This leads to the nuclear export of BACH1 into the cytosol, where it is degraded via the proteasome. In turn, this allows Nrf2 to form a Nrf2-sMaf dimer at the enhancer regions and induce *HMOX1* transcription [37,38,39,40]. 

Although Nrf2 and BACH1 are the major regulators of *HMOX1* expression, other mechanisms may also be relevant depending on the cellular context. The transcription factors AP-1, NF-κB, and hypoxia-inducible factor-1 (HIF-1α) have been described to induce *HMOX1* transcription [41,42,43,44]. AP-1 stimulated the expression of *HMOX1* through the cooperation of AP-1 subunits with various transcription factors, including Nrf2 in human endothelial cells [41,42,45]. HIF-1α, a heterodimeric transcription factor induced by low oxygen levels, stimulated *HMOX1* expression in human microvascular endothelial cells and in decidual stromal cells, probably via Nrf2 [43,46]. NF-κB was shown to stimulate *HMOX1* expression by binding to the *HMOX1* promoter in mouse cardiac tissue [44]. Furthermore, the involvement of NF-κB and AP-1 in *HMOX1* activation was observed in neonatal ventricular myocytes after hypoxia induction [47].

### 3.2. Regulation of HMOX1 Expression by Signaling Pathways and Post-Transcriptional Mechanisms

In addition to direct upregulation of *HMOX1* via transcription factors, indirect stimulation via signaling pathways triggered by external stimuli has also been described. Several signaling pathways involved in the cellular stress response have been implicated in the regulation of *HMOX1* transcription [48,49,50,51]. For example, extracellular stimuli can affect *HMOX1* expression via mitogen-activated protein kinases (MAPKs), including extracellular signal-regulated kinase (ERK), c-Jun N-terminal kinase (JNK), and p38 MAPK, which further target transcription factors [48,49,50]. In human hepatocyte L-02 and HepG2 cells, phosphorylation of ERK and p38 has been shown to stimulate *HMOX1* transcription via increased Nrf2 expression and nuclear translocation after treatment with either diallyl sulfide or floridoside [48,49]. Furthermore, protein kinase C (PKC) is capable of phosphorylating Nrf2 in response to oxidative stress. This posttranslational modification of Nrf2 promotes its dissociation from KEAP1 and allows Nrf2 to translocate into the nucleus, where it induces *HMOX1* expression [52]. Moreover, Signal Transducer and Activator of Transcription (STAT)-3 and phosphatidylinositol 3-kinase (PI3K)-dependent activation of *HMOX1* expression in human and mouse myeloid cells by IL-10 has been described [53]. In murine brain cells, PI3K activation was also found to primarily promote translocation of Nrf2 to the nucleus [54]. Furthermore, the enzyme AMP-activated protein kinase (AMPK) stimulates *HMOX1* expression by increasing the nuclear localization and phosphorylation of Nrf2 in vivo and in various human epithelial cell lines. AMPK is a serine/threonine protein kinase and energy sensor that is activated by high AMP:ATP ratios as a result of various cellular and metabolic stressors such as hypoxia, ischemia, or heat shock [55,56]. 

Several studies also indicated that *HMOX1* is post-transcriptionally regulated by microRNAs (miRs) [57,58,59,60,61]. The non-coding RNAs showed direct effects on *HMOX1* expression in neurons, which was increased by miR-494 under oxidative conditions in a BACH1-independent manner [57]. In contrast, binding of miR-24 or miR-200c to *HMOX1* suppressed its expression in human smooth muscle cells [62] and in proximal tubule cells treated with ochratoxin-A [58]. Moreover, control of upstream regulators of *HMOX1* by miRs may indirectly affect *HMOX1* expression. Whereas miR-153 inhibited the expression of Nrf2 in rats after ischemia-reperfusion (IR) [59], miR-101 increased Nrf2 expression by inhibiting Nrf2 ubiquitination in human umbilical vein endothelial cells (HUVEC) under hypoxic conditions [63]. Moreover, downregulation of BACH-1 translation in HUVECs by miR-155 [60] and degradation of *Keap-1* mRNA in breast cancer cells by miR-200a indirectly increase *HMOX1* expression [61].

## 4. Other HO Isoforms

Besides HO-1, which is inducible by the transcription factor Nrf2 as outlined above, another isoform termed HO-2 exists. HO-2 is encoded by *HMOX2* located on chromosome 16 (16q12) [24] and gives rise to a 36 kDa protein. It is constitutively expressed in neurons and is also found in other tissues such as the spleen and myocardium [64]. HO-2 breaks down hemin with similar efficiency as HO-1, but it possesses additional heme-regulatory motifs (HRMs) that bind heme and transfer it to the catalytic center of HO-2 [65]. The two HRMs contain cysteine residues that form a disulfide bond; when reduced, these cysteines are available to bind hemin [65]. In total, HO-2 has three heme binding sites: one at its catalytic site and the others at its two HRMs [64].

The activity of HO-2 is regulated by posttranslational modifications. Activation of PKC leads to phosphorylation of casein kinase 2 (CK2), which in turn activates HO-2 through phosphorylation at Ser79 [66]. Currently, only one specific chemical activator of HO-2 is known. Menadione (Vitamin K3) has no effect on HO-1 but can increase the enzymatic activity of HO-2 by up to 30-fold in in vitro models [67]. HO-2 attracted attention due to its various beneficial effects within cells and organs such as the liver, testis, and brain, which express it constitutively. In the testis, HO-2 is involved in the modulation of the ejaculatory activity [68]. In contrast, HO-2 plays a dual role in the brain, which leads on the one hand to antioxidant and anti-inflammatory effects because of products generated by heme catabolism [69,70]. On the other hand, prooxidant and cytotoxic effects are observed due to abundant formation of iron after intracerebral hemorrhage (ICH) [71]. Furthermore, HO-2 is able to suppress inflammatory pathways through the reduction of pro-inflammatory cytokines, such as IL-1b, TNFα and interleukin 6 (IL-6), in macrophages [72]. Many potential therapeutic applications with regard to HO-2 are investigated like preventing renal injury and diabetes [73]. HO-2 was also shown to be involved in the onset of neurodegenerative diseases owing to an *HMOX2* polymorphism. This leads to iron release and abnormal iron deposition, and subsequently Alzheimer’s disease, where overexpression of *HMOX2* was found [74,75].

A third isoform, HO-3, has been described in rats. It has been identified as a pseudogene processed from HO-2 transcripts, but its function is still unclear [76].

## 5. Cytoprotective Effects of HO-1

**Figure 4:**
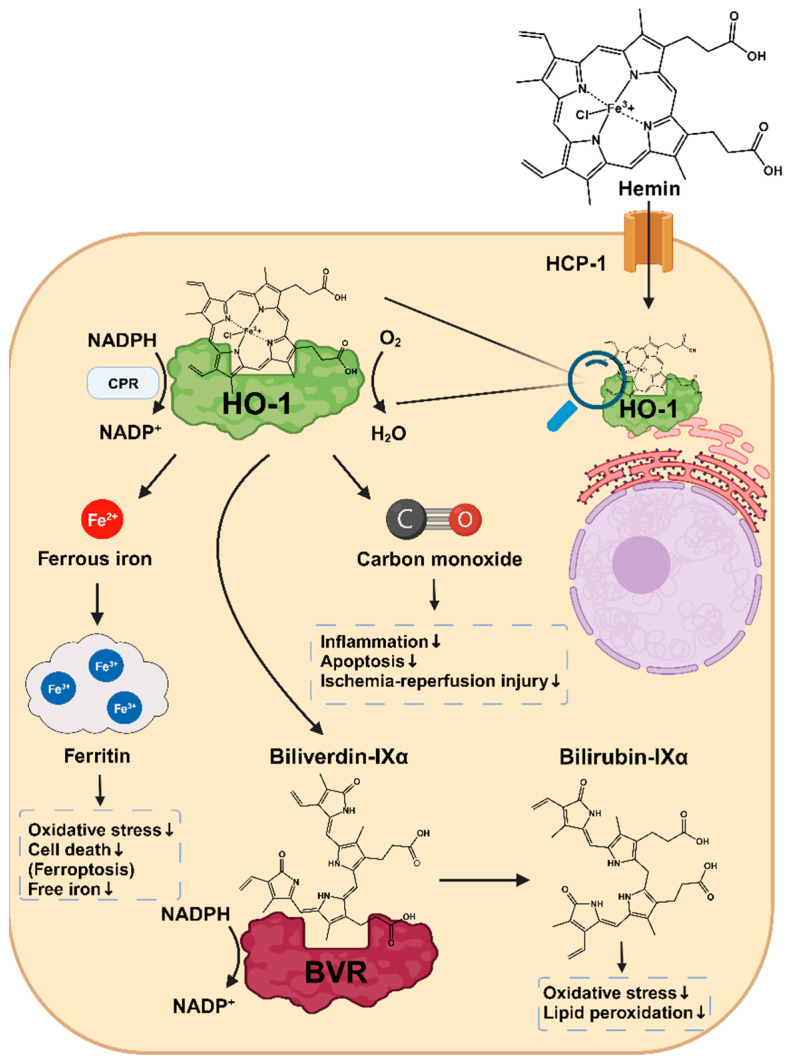
Cytoprotective effects of HO-1 and its breakdown products. Ferric heme (hemin) is internalized into the cell via heme carrier protein 1 (HCP-1). Intracellular ferric heme is degraded in the cytosol by heme oxygenase 1 (HO-1) located at the endoplasmic reticulum (ER). This reaction requires an electron transfer by NADPH-cytochrome P450 oxidoreductase (CPR), which catalyzes the conversion of NADPH and O_2_ into NADP^+^ and H_2_O. The released ferrous iron (Fe^2+^) is oxidized to ferric iron (Fe^3+^) by ferritin and then stored. The protoporphyrin ring is broken down to carbon monoxide (CO) and biliverdin-IXα, which is further converted to bilirubin-IXα by biliverdin reductase (BVR). HO-1 as well as its degradation products CO and biliverdin display multiple cytoprotective and antioxidative functions (please refer to the main text for further details). Created with BioRender.com, accessed on 25 October 2023.

Heme, the substrate of HO-1, is an organic, iron-containing protoporphyrin ring that is essential for numerous biological processes [77,78]. As a prosthetic group of several proteins, heme plays an important role in oxygen transport (e.g., hemoglobin and myoglobin), oxidative metabolism (e.g., catalases, oxygenases), detoxification of xenobiotics (cytochrome P450 enzymes), electron transfer (cytochromes a, b, c), and microRNA processing (DGCR8) [77,79]. On the other hand, free heme is a known catalyst of radical formation, a pro-pathogenic mediator, and a pro-oxidant with cytotoxic effects [2,21,80]. It also induces lipid peroxidation and chronic inflammation [81,82]. The degradation of heme by HO-1 contributes to the maintenance of intracellular homeostasis and represents a direct cytoprotective effect of HO-1. Studies in HO-1-deficient mouse models as well as several cases of HO-1 deficiency in humans caused by *HMOX1* mutations highlight the importance of HO-1 for tissue iron and organ homeostasis, protection against oxidative stress, and macrophage function [14,83,84]. The heme degradation products BV/BR and CO have additional cytoprotective effects, which will be detailed below [72,85,86,87,88,89,90,91,92] (Figure 4).

BV and BR are potent antioxidants that show cellular protective effects in vitro and in vivo [72,87,88]. Both BV and BR protected EA.hy926 cells, a standard model of human vascular endothelium, from oxidative stress with an EC_50_ value equivalent to BR serum concentrations [87]. The protective effects of BV and BR are likely due to the biliverdin-bilirubin redox cycle, in which BR is oxidized to BV by scavenging oxidants. BVR then catalyzes the NADPH-dependent reduction of the generated BV back to BR [85]. The reduction of BV to BR is essential for its antioxidant activity, which was demonstrated in a BVR knockout model. In *BVR^−/−^* mice, 100-fold higher BV levels were measured, while BR levels were 25-fold decreased. This was associated with increased serum levels of oxidative stress [72]. BR protects against lipid peroxidation in a more potent manner than the well-known antioxidant glutathione [88]. A study in HeLa cells revealed a protective effect for BR up to a 10,000-fold excess of H_2_O_2_, while the depletion of BVR by small interfering RNA (siRNA) led to a significant increase in tissue ROS levels and cell death [85,88]. Moreover, high BR levels negatively correlate with oxidative stress outcomes such as diabetic complications, chronic inflammation, and cardiovascular disease such as atherosclerosis [86,93,94,95]. Zhu and colleagues summarized in a meta-analysis that high BR levels are associated with a protection against diabetic nephropathy, retinopathy, and neuropathy, as well as other diabetic complications, for which oxidative stress is considered as a major cause [93]. A cohort study involving 4196 men and 4648 women also found an association between high BR levels and reduced cardiovascular mortality [86]. In mice with low-density lipoprotein receptor deficiency, BR has also been shown to inhibit atherosclerotic plaque formation by scavenging ROS signaling intermediates, thereby impairing vascular cell adhesion protein 1 (VCAM-1)- and intercellular adhesion molecule 1 (ICAM-1)-mediated endothelial leukocyte migration [94].

While exogenous CO has long been known as a toxic gas, its potential for treating various medical conditions is increasingly recognized [96,97,98]. Endogenous CO is mainly formed due to the degradation of heme by HO-1 [99]. It plays an important physiological role in neuronal signal transduction as well as in the maintenance of vascular tone [97,100,101]. It also exhibits anti-apoptotic, anti-inflammatory, and anti-proliferative effects, thus protecting tissues [89,90,92,96,102,103,104]. In this context, CO acts as a modulator of a variety of signaling pathways, which are detailed below.

CO can downregulate NADP(H)-oxidase-dependent ROS formation in macrophages, thereby inhibiting TLR2,4,5 and 9 signaling pathways. The resulting suppressed migration of TLRs to membrane rafts decreases cytokine production and negatively regulates the proinflammatory cascade [104]. On the other hand, CO can also trigger increased mitochondrial ROS formation in macrophages by inhibiting cytochrome c oxidase. Due to this inhibition, the LPS-induced inflammatory response in macrophages was limited [89]. Such a macrophages-derived ROS burst also leads to an increase in peroxisome proliferator-activated receptor-γ (PPARγ), a nuclear hormone receptor that plays an important role in the expression of genes involved in inflammatory signaling pathways in macrophages [105]. In a mouse model of acute lung injury, CO suppressed the expression of a key inflammatory mediator, early growth response protein 1 (Egr-1), and reduced tissue damage. Inhibition of PPARγ abolished both effects [105]. Via activation of the MKK3/p38β-MAPK pathway, CO triggered anti-inflammatory effects in LPS-induced inflammation models. While the expression of pro-inflammatory cytokines such as TNF-α, IL-1β and macrophage inflammatory protein-1β (MIP-1β) was decreased, there was a concomitant increase in the production of the anti-inflammatory cytokine IL-10 [106]. The anti-inflammatory effect of HO-1/CO was also demonstrated in murine colitis models, in which overexpression of HO-1 as well as inhalation treatment with CO led to a decrease in the pro-inflammatory cytokines TNF-α and interferon-γ (IFN-γ) and tissue-associated myeloperoxidase (MPO) activity in the colonic mucosa [90,107].

In addition to the described anti-inflammatory effects, CO-dependent induction of the p38 MAPK pathway also protects against spontaneous and induced apoptosis [91,96,102,108,109,110]. IR injury is common in organ transplantation and can be attenuated by HO-1 induction via cobalt protoporphyrin IX (CoPP) and exogenous CO administration [96,108,109,110]. CO protected endothelial cells from TNF-α-mediated apoptosis by increasing TNF-β-dependent anti-apoptotic genes such as *c-IAP2* in a p38 MAPK-dependent manner [102]. Moreover, CO-induced activation of the MKK3/p38α-MAPK pathway led to the downregulation of *Fas ligand (FasL)* and suppression of caspase-8 activity in primary rat pulmonary artery endothelial cells and in a mouse lung IR model [108]. Furthermore, an increased expression of anti-apoptotic Bcl-2 family proteins such as Bcl-2 and Bcl-X_L_ with concomitant reduction in expression of pro-apoptotic proteins such as Bid was observed, ultimately preventing release of cytochrome c from mitochondria and thus protecting against IR-induced apoptosis [108]. Similar protective effects of CO against IR-induced apoptosis were also described in lung epithelial cells, which was attributable to STAT3 inhibition via PI3K/Akt- and p38 MAPK-dependent pathways [109].

CO-induced mitochondrial ROS formation through inhibition of NADPH oxidase, as described previously, also leads to a decrease in ERK1/2 MAPK phosphorylation and cyclin D1 expression, which allowed CO to inhibit proliferation in airway smooth muscle cells [92]. In addition to its anti-proliferative effect, CO may also contribute to re-endothelialization and prevent neointima formation after carotid artery balloon injury in male rats. Moreover, proliferation, adhesion and migration of HUVEC were promoted by exogenous CO in a PI3K/Akt/eNOS pathway-dependent manner [111].

Last but not least, the HO-1 breakdown product Fe^2+^ increases the synthesis of the iron storage protein ferritin [112,113]. Ferritin consists of two subunits, Ferritin heavy-chain (FtH) and Ferritin light-chain (FtL), which have different functions. FtH has a ferroxidase center where Fe^2+^ is oxidized to the less reactive Fe^3+^. FtL helps to release Fe^3+^ from the ferroxidase center and to store it [114]. Several studies indicated the protective role of ferritin against free iron, oxidative stress, and cell death [115,116,117]. In mice with intestinal *FtH* deletion, intestinal ferritin has been shown to be essential for regulating iron absorption and thus protecting the body from excessive iron levels [115]. In a model of acute kidney injury (AKI), mice with *FtH* deletion in the proximal renal tubule showed significantly increased mortality, apoptosis rate, and worsening of functional and structural renal injury associated with elevated HO-1 levels. It was also shown that only FtH proficient mice showed an increase in the iron transporter ferroportin (FPN) after glycerol-induced rhabdomyolysis, resulting in an increased export of deleterious ferrous iron across the basolateral membrane into the circulation. These observations suggest that ferritin also promotes the cellular export of toxic free iron, thereby protecting cells [116]. 

In summary, HO-1 has manifold cytoprotective functions. In addition to the degradation of cytotoxic free heme, the degradation products CO and BV/BR as well as ferritin induction by the released Fe^2+^ contribute to cell protection via multiple mechanisms. Depending on the tissue and cell type, both the activated signaling pathways and the resulting effects may vary [72,90,91,92,96,111].

## 6. Involvement of HO-1 in Intestinal Inflammation and Chronic Diseases

**Figure 5:**
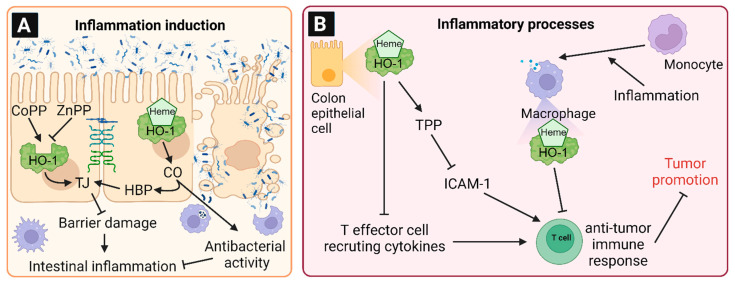
Role of HO-1 in prevention of an inflammation response due to intestinal epithelial barrier damage (**A**) and regulation of inflammatory processes (**B**). Depending on the cell-type specific HO-1 expression the anti-tumor immune response is boosted or inhibited. HBP: heme binding protein; ICAM-1: Intercellular adhesion molecule 1; TJ: Tight junction; TPP: tristetraprolin. Created with BioRender.com, accessed on 26 October 2023.

Intestinal inflammation is mainly triggered by disruption of the epithelial barrier function, followed by infiltration of bacteria or bacterial components [118]. It was reported that free heme induced barrier disruption in filter-grown, differentiated human epithelial colorectal adenocarcinoma cells (Caco-2) [18]. This effect was attenuated by induction of HO-1, whereas inhibition of HO-1 resulted in enhanced barrier disruption [18]. In CCl_4_-treated mice, pathological changes of the colon with reduced colon length, lower levels of tight junction (TJ) proteins and higher TNFα levels were observed, which was influenced by intestinal HO-1 expression (Figure 5A) [118]. CoPP-induced HO-1 expression attenuated the changes of TJ proteins and TNFα levels. In contrast, inhibition of HO-1 by ZnPP increased the pathological changes [118]. The protective effects of HO-1 induction were traced back to CO-releasing molecule-2 (CORM-2) [118]. Due to its high affinity, the heme breakdown product CO can bind to heme-binding proteins that form the tight junction complex, which is crucial for the cellular barrier function, and re-close the barrier by altering protein configuration [119]. Similar to CCl_4_-treated mice, the induction of HO-1 or CO release by CORM-2 improved intestinal barrier function in rats suffering from cholestasis [120].

Inflammatory bowel disease (IBD) describes a group of chronic inflammatory diseases of the intestinal tract that includes ulcerative colitis (UC) and Crohn’s disease (CD), which show an increasing incidence worldwide [121,122]. A comparison of diseased and healthy intestinal tissue from UC patients revealed an increased HO-1 expression on mRNA and protein level in the inflamed tissue. These observations suggest an important role of HO-1 in IBD [123]. In several IBD models, it was shown that expression of HO-1 as well as exposure to CO resulted in an amelioration of colitis [19,124]. CO suppressed Th17-induced colitis in *Il10*^−/−^ mice via a HO-1-dependent mechanism. Treatment with CO or the HO inducer CoPP ameliorated chronic intestinal inflammation in T cell receptor-alpha (TCR-α)-deficient mice, in which colitis is driven by Th2 cytokines, similar to human UC. The decrease in intestinal inflammation occurred through inhibition of colonic interleukin-1β (IL-1β), TNF, and IL-4 production and increased IL-10 secretion [124].

In line with these findings, HO-1-dependent reduction of inflammatory processes were reported in HO-1 knockout mice and HO-1-deficient human subjects. Mouse studies with *Hmox1^+/+^*, *Hmox1^+/−^* and *Hmox1^−/−^* genotypes revealed a stronger sensitivity of *Hmox1^−/−^* mice towards LPS as inflammatory stimulus, resulting in significantly higher cytokine release and a survival rate of only 7%. In contrast to that, the survival rates of *Hmox1^+/+^* and *Hmox1^+/−^* mice were 100% and 94%, respectively [15]. Similar results with a 37-fold increase in IL-6 levels were seen in LPS-treated *Hmox1* knockout mice [125]. Case reports of human HO-1-deficient patients showed chronic inflammation and significantly higher ROS-related effects than healthy individuals [83]. Greil and colleagues presented the case of a Turkish male patient with a loss of function mutation in *HMOX1* [126]. The patient showed high levels of ROS-induced acrolein-lysine and 8-oxoguanine, an important marker of oxidative DNA damage. Moreover, peripheral blood mononuclear cells obtained from this patient were more sensitive to oxidative stress inducers and inflammatory stimuli [126].

Furthermore, several studies suggest that both the composition of the gut flora and the patient’s protective mechanisms play a crucial role in the development of IBD [127,128]. Colonization of germ-free wild-type (*Il10^+/+^*) and *Il10*^−/−^ mice with a specific pathogen-free (SPF) microbiota resulted in increased *TNF* and *Il12b* expression as well as enhanced colonic inflammation in *Il10*^−/−^ mice. In contrast, increased *Hmox1* expression in the colon and elevated colonic HO-1 protein levels were observed in WT animals as compared with *Il10*^−/−^ animals. Further Nrf2-regulated genes like *Fth1* were also highly expressed in WT, but not *Il10*^−/−^ mice. Pharmacological induction of HO-1 with CoPP in germ-free *Il10^−/−^* mice before colonization with SPF microbiota resulted in amelioration of colitis and decreased secretion of IL-12 p40. These observations suggest a protective induction of HO-1 by the microbiome in a Nrf2- and TLR-dependent manner. Furthermore, the treatment of *Il10*^−/−^ mice with CoPP and the CO-releasing molecule ALF186 resulted in increased bactericidal activity of macrophages against *E. coli*, *E. faecalis*, and *S. typhimurium* in addition to bacterial elimination in mesenteric lymph nodes. This suggests that the anti-inflammatory effect of HO-1 and CO is due to the increased bactericidal activity of macrophages, which are thus better able to eliminate bacteria that may cause or exacerbate colonic inflammation [129]. This hypothesis was later verified by in vitro studies using bone marrow derived macrophages from *Hmox1*-deficient mice. CO significantly increased the bacterial killing by macrophages and counteracted the knockout-derived defect [20]. 

Therefore, HO-1 expression in intestinal epithelial cells as well as in macrophages is important to prevent and suppress inflammation, while the time point of HO-1 induction seems to be critical (Figure 5B). Interestingly, HO-1 induction by CoPP prior to colitis induction by dextran sodium sulfate (DSS) reduced the histological degree of intestinal inflammation [130]. It should be emphasized that such an anti-inflammatory effect was only observed, if the HO-1 expression was increased as a preventive treatment, not as a therapeutic approach after the onset of inflammation. The authors hypothesized an HO-1 dependent lower expression of ICAM-1 as a key anti-inflammatory effect. ICAM-1 is an adhesion protein, responsible for the regulation of cell—cell interactions like lymphocyte-mediated cytotoxicity in tumor cells, which is therefore important for the immune surveillance system [131,132]. In this context, HO-1 induction in response to hemin exposure reduced ICAM-1 expression in HT29 and Caco2 CRC cells by regulation of the RNA-binding protein tristetraprolin (TTP). Moreover, hemin reduced the expression of T-effector cell-recruiting cytokines, which further led to anti-inflammatory effects [131]. ICAM-1 is considered as potential oncogene in colorectal carcinogenesis, since it promotes the migration, invasion and expression of epithelial-mesenchymal transition markers [133]. Thus, ICAM-1 could represent a possible link between HO-1 and tumor progression (see Section 9).

## 7. Colorectal Carcinogenesis and the Role of HO-1

Colorectal cancer (CRC) is one of the most frequent tumor entities worldwide, with an increasing incidence in adults under the age of 50 years [134,135]. CRC is causally linked to genetic predisposition, chronic intestinal inflammation, lifestyle and nutritional risk factors [135,136,137,138]. Sporadic colorectal carcinogenesis is often illustrated with the model developed by Fearon and Vogelstein, which describes a characteristic series of mutations in tumor suppressor genes and proto-oncogenes [139]. The formation of CRC is a multi-step process occurring over decades [140]. The first step is called tumor initiation, in which normal intestinal epithelial cells acquire mutations in critical genes, i.e., tumor suppressor genes and/or proto-oncogenes. These mutations can be induced by various environmental or dietary genotoxins, such as heterocyclic aromatic amines and *N*-nitroso compounds [21,81,141,142,143]. Furthermore, hereditary or acquired DNA repair defects increase the risk that spontaneous or induced DNA damage in intestinal epithelial cells is fixed into mutations. With regard to CRC formation, defects in mismatch repair, but also *O*^6^-methylguanine-DNA methyltransferase (MGMT) and MYH, are of particular relevance [144,145,146]. Mutations in the WNT signaling pathway as the pivotal regulator of intestinal epithelial cell turnover causes hyperproliferation and promotes the formation of aberrant crypt foci [139,140]. These mutations are mainly found in the tumor suppressor gene *APC*, but also observed in its downstream target *ß-catenin* [147]. In the next stage termed tumor promotion, a higher cell proliferation together with, e.g., inactivated DNA repair genes such as *MGMT*, increases the probability for further mutations thereby activating proto-oncogenes such as *Kirsten-ras (KRAS)* and inactivating tumor suppressor genes like *TP53* [139,148,149]. This results in the formation of a benign adenoma. Subsequently, the benign tumor evolves into a malignant carcinoma, with a progressive accumulation of further mutations and epigenetic alterations [139]. At this stage called tumor progression, the tumor grows independently, displaces normal healthy tissue and spreads to distant parts of the body via blood and lymph vessels. This process is called metastasis and is triggered by epithelial-mesenchymal transition (EMT) of cancer cells, whereby the cells are able to migrate and invade into surrounding and distant tissue [150].

In colon (cancer) tissue, HO-1 seems to be involved in tumor induction and progression due to its stage-specific expression. Several independent studies detected a significantly higher HO-1 expression and activity in tumor tissue from CRC patients than in adjacent normal colon tissue [151,152,153]. Moreover, well-differentiated tumors showed the highest HO-1 expression, whereas the content in moderately and poorly differentiated tumors were lower [153]. Concomitant with the overall increase in HO-1 expression, its localization altered during carcinogenesis, with stage-dependent higher nuclear HO-1 levels in polyps and adenocarcinoma as compared to non-tumor tissue [151]. Nuclear localization of HO-1 was also observed in human HCT116 CRC cells. In both untreated and hemin-treated cells, HO-1 localized to the nucleus, while hemin increased the HO-1 expression without affecting its subcellular localization [151]. One possibility for the nuclear translocation of HO-1 might be its passive diffusion through the nuclear pore complex, which has a size limit for proteins with more than 40–60 kDa [154,155]. Furthermore, nuclear localization sequences were identified in HO-1, which can mediate its translocation [156]. Nuclear translocation of HO-1 in mouse fibroblasts and hepatoma cells were attributable to the cleavage of a 52-amino-acids-spanning region from its C-terminus [9]. The truncated HO-1 in the nucleus showed lower enzymatic activity, but mediated the activation of transcription factors involved in the protection from oxidative stress and in cell proliferation, such as AP-1, AP-2 and Brn-3, by alteration of their DNA binding activity [9]. If this specific mechanism is also relevant for colon (cancer) cells, it is not yet known. Interestingly, in non-cancerous human colonic epithelial cells (HCEC), hemin induced the expression of HO-1 in a concentration-dependent manner, which was however exclusively localized in the cytoplasm [21]. To sum up, the expression and localization of HO-1 in colon tissue seems to be associated with CRC formation and might be useful as diagnostic and prognostic marker. In line with this notion, a study pointed out that the HO-1 degradation product CO, which is bound to hemoglobin (COHb) and circulates in the bloodstream of patients, might be a diagnostic marker. The COHb levels in CRC patients were significantly higher than in non-cancer patients [153]. The suitability of COHb as diagnostic marker is supported by a strong correlation of increased serum COHb levels, tumor growth, and HO-1 activity in tumor-bearing mice [153]. 

While nuclear HO-1 expression positively correlates with CRC formation, it might also influence the survival rate of patients. CRC patients, whose tumor tissue was stained positive for HO-1, showed a significantly higher survival rate as compared to HO-1-negative cancer patients [151,157]. Furthermore, tumors with high HO-1 expression levels were associated with lower lymphatic invasion. Thus, the increased HO-1 levels may be a protective mechanism to suppress colorectal carcinogenesis. In contrast to these studies, a high HO-1 expression in macrophages near colorectal tumors correlated with lower patients’ survival rates as well as higher risks for lymph node metastasis [158]. Alaluf and coworkers demonstrated a strong HO-1 induction in mice as part of the differentiation of monocytes to macrophages by the tumor microenvironment, e.g., the inflammation of gut mucosa. Furthermore, HO-1 expression by these tumor-associated macrophages led to significant immunosuppressive activity, which reduced the T-cell mediated anti-tumor immune response. In turn, HO-1 inactivation enhanced the anti-tumor cell response and suppressed the HO-1-mediated tumor-promoting effects of macrophages [159]. Taken together, the HO-1 expression in colon epithelial cells may reduce tumor formation, while high HO-1 levels in tumor-associated macrophages seem to promote carcinogenesis (see also Figure 5B).

Similar to inflammation and immune reaction, a clear correlation of HO-1 and miR expression was seen in CRC tissue. HO-1 expression is suppressed by its negative regulator BACH1 [29,31], which occurred to a significantly higher extent in non-tumor colon tissue than in cancer tissue [160]. It was hypothesized that the reduction of BACH1 expression during carcinogenesis might be regulated by an increase in miR-135a, because of a documented negative correlation of BACH1 and miR-135a expression in colon (cancer) cells [160]. The underlying mechanism of BACH1 regulation by miR-135a is still unknown. However, the lower BACH1 expression could be mainly responsible for the higher HO-1 levels in colon cancer tissue [160]. In accordance with HO-1, the miR-135a expression is higher in CRC tissue and increases with the CRC tumor stage [151,152,153,160,161]. This was also observed in the CRC cell lines SW480 and SW620, in which the miR-135 expression was higher than in normal mucosa tissue samples [161]. The CRC cell lines were analyzed in more detail by reduction of the miR135a expression using the synthesized oligonucleotide inhibitor antagomiR-135a. The miR-135a-induced promotion in tumor progression-related processes was reduced likewise [161]. Migration and cell invasion are crucial steps in EMT, an important process for tumor progression and metastasis [150,162]. Inhibition of miR-135a expression in CRC cell lines significantly decrease their ability of migration, invasion and proliferation [161]. These cellular properties are relevant for CRC formation and are often accompanied by high HO-1 expression in colon tumor tissue due to reduced cellular BACH1 levels. Cell invasion and anchorage-independent growth of CRC cells can be further directly induced by HO-1 overexpression. In CRC patient tissue the expression of the endothelin-converting enzyme-1 (ECE-1) was significantly higher than in normal tissue and correlates with HO-1 expression [163]. ECE-1 regulates cancer stem cell properties, which was shown in vitro by HO-1 or ECE-1 overexpression [163,164]. In both studies, the transfected cells showed a higher expression of CD44 and CD133 [163,164], known as cancer stem cell markers and essential in tumor progression [165,166,167]. Moreover, high HO-1 or ECE-1 expression increased the formation of spheres and its metastatic potential [163,164]. 

These studies with human CRC cell models and colon (cancer) tissue samples provided first insights, in which processes HO-1 is involved, with both anti-tumorigenic and tumor-promoting effects described. In the following two chapters, the role of HO-1 in colorectal tumor induction and progression is discussed in more detail. 

## 8. Role of HO-1 in Tumor Induction

### 8.1. HO-1-Dependent Protection against ROS and Lipid Peroxidation

**Figure 6:**
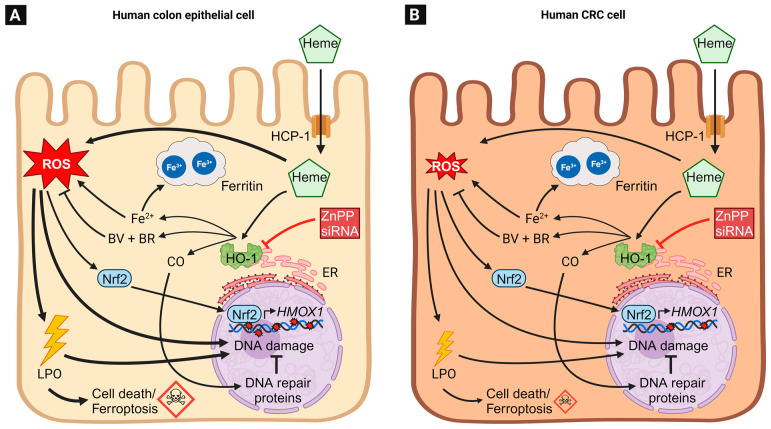
HO-1-dependent protection against heme-induced oxidative stress and genotoxicity in human colon epithelial cells (**A**) and human CRC cells (**B**). Non-cancer colon epithelial cells are more sensitive to heme toxicity, showing higher levels of reactive oxygen species (ROS), lipid peroxidation (LPO) and DNA damage. BR: bilirubin; BV: biliverdin; ER: endoplasmic reticulum; HCP-1: heme carrier protein-1; Nrf2: nuclear factor erythroid 2-related factor 2. Created with BioRender.com, accessed on 8 November 2023.

ROS can be involved in tumor formation, e.g., by induction of DNA damage or by other mechanisms like the regulation of immune cells [168]. There is a close relation of ROS and HO-1 expression (summarized in Figure 6). On the one side, the induction of oxidative stress and ROS by H_2_O_2_, growth factors or heme, led to higher HO-1 expression in non-tumor and tumor colon cell lines [21,169]. Furthermore, HO-1 was also shown to be upregulated in colon epithelium of mice exposed to dietary heme iron, whereas no induction of HO-1 was observed in the control group receiving non-heme iron [81]. As described in Section 3, ROS induces the release of Nrf2 from the KEAP1-Cul3 complex, followed by phosphorylation and nuclear translocation of Nrf2. In the nucleus, Nrf2 binds to ARE in DNA to upregulate the transcription of genes involved in the antioxidant response like *HMOX1* [31,35,36]. On the other side, HO-1 reduces the cellular ROS level by generation of BV and BR, which are known antioxidants by reacting with ROS and lipid peroxidation products [85,170,171]. In line with that, the chemical inhibition of HO-1 activity with ZnPP led to significantly higher ROS levels in treated CRC cells [172,173,174]. In Caco-2 cells, HO-1 expression was increased by both heme and nitrosylated heme in a comparable manner [141], indicating that both heme compounds independent of their ligand are substrates for HO-1.

As mentioned above, the interrelation of heme, ROS and HO-1 was shown for both non-tumor as well as CRC cells. Nevertheless, non-malignant HCEC were more sensitive to hemin than CRC cells (HCT116 cells and others) as demonstrated by higher ROS levels, oxidative DNA damage, and cytotoxicity [21]. The inhibition of HO-1 by ZnPP or its siRNA-mediated knockdown further increased the hemin-induced ROS formation and toxicity. HCT116 CRC cells were characterized as a non-differentiated cell line, classified as stage 3 of the TNM system [175,176] with low basal HO-1 levels, which is in accordance with the above-mentioned association of highly differentiated tumors with higher HO-1 expression levels [153]. Moreover, poorly differentiated colon cell lines like COLO205, HCT-15 and LOVO were more sensitive towards H_2_O_2_ as compared to the well-differentiated cell line HT-29 [177]. Unfortunately, HO-1 expression was not analyzed in this setup.

### 8.2. Role of HO-1 in Genotoxicity and DNA Damage Response

**Figure 7:**
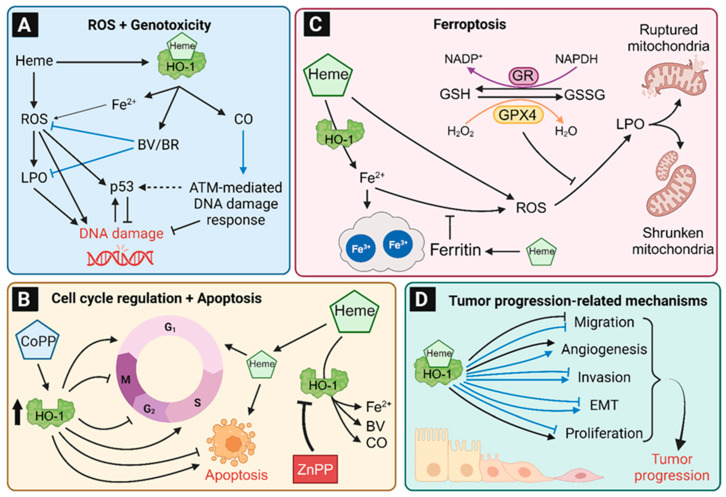
Impact of HO-1 in cancer-related mechanisms. (**A**) The protective role of HO-1 in ROS formation and reduction of genotoxic effects. (**B**) Regulation of the cell cycle and apoptosis by HO-1. (**C**) Contribution of HO-1, ferritin and antioxidant enzymes in ferroptosis. (**D**) Induction and inhibition of tumor progression-related mechanisms. Blue arrows and lines indicate mechanisms, which were documented in non-colon cancer cell lines; ATM: ATM serine/threonine kinase; BR: bilirubin; BV: biliverdin; GPX4: glutathione peroxidase 4; GR: glutathione reductase; GSH: glutathione; GSSG: glutathione disulfide; LPO: lipid peroxidation; p53: Tumor protein p53; ROS: reactive oxygen species. Created with BioRender.com, accessed on 25 October 2023.

HO-1-derived heme degradation mainly prevents high cellular ROS levels, but it also suppresses lipid peroxidation and DNA damage as biological consequences (Figure 7A) [21,178,179]. Cellular lipid hydroperoxides were formed by the reaction of (e.g., heme-induced) ROS with the unsaturated fatty acids of lipids and can further induce DNA damage, such as DNA adducts and DNA strand breaks [180,181,182]. Several studies using normal or CRC cell lines showed that the HO-1 inhibition by ZnPP alone, as well as in combination with genotoxic stress, increases DNA damage [21,174,183]. Li and colleagues analyzed the influence of HO-1 inhibition on the toxic effects of the chemotherapeutic paclitaxel. Upon inactivation of HO-1 by ZnPP, the CRC cells displayed more DNA damage, as analyzed by γH2AX levels, followed by higher apoptosis and lower cell viability [183]. These effects were already seen for ZnPP without paclitaxel treatment, whereby a combination treatment significantly increased the effects. Thus, the inhibition of HO-1 sensitizes CRC cells for the chemotherapeutic effects of paclitaxel. Another important factor in genome protection by HO-1 is the activation of DNA repair by the heme degradation product CO. HO-1 deficient mice displayed significantly higher levels of γH2AX-positive cells in the kidney, lung, liver and spleen [184]. As mentioned above, yH2AX is an established marker for DNA damage [185,186]. Furthermore, wild-type mice were exposed to CO and showed significantly lower levels of doxorubicin-induced DNA damage in the intestine as well as in the kidney, liver, and lung [184]. A follow-up cell culture study in prostate and kidney cancer cells provided evidence that CO increased the levels of phosphorylated DNA repair proteins like p-BRCA1 and its upstream DNA damage response kinases p-ATM and p-ATR. ATM was revealed as the most important factor in HO-1 and CO-induced DNA repair, since the beneficial effects were abrogated by the ATM inhibitor KU-55933 [184]. Finally, HEK293 kidney tumor cells with HO-1 knockout were analyzed in detail. The HO-1 deficient cells showed significantly lower levels of p-ATM, p-ATR, p-BRCA1, and p-CHK2. Therefore, CO generated by heme breakdown seems to activate DNA double strand break repair with ATM as essential regulator [184]. 

### 8.3. Regulation of the Cell Cycle, Apoptosis and Cell Viability by HO-1

ROS formation and DNA damage occur at any time in our cells, while it is hypothesized that each cell produces 50 hydroxyl radicals per second [187]. If the damage levels exceed the cellular DNA repair capacity, apoptosis is induced [188]. Thus, apoptosis is an essential mechanism to prevent carcinogenesis by elimination of initiated cells via controlled cell death induction [188,189]. Interestingly, this tumor-protective mechanism can be affected by HO-1-regulating compounds like CoPP and ZnPP [174,190]. The impact of HO-1 in apoptosis and cell viability was shown in several colon cell lines as well as in mouse studies performed in independent experiments. However, these results are inconsistent and the role of HO-1 in apoptosis is not yet clarified (Figure 7B). The induction of HO-1 expression by CoPP increased the S-population and reduced the G2/M-population of Caco-2 cells, while apoptosis was reduced likewise [190]. Similar results were seen in murine C-26 cells, in which a high HO-1 expression dose-dependently protected the cells against apoptosis [191]. In accordance with these findings, CoPP treatment in mice with DSS-induced colitis led to less apoptotic cells in the colon as compared to untreated animals [130]. While HO-1 induction decreased apoptosis, its inhibition by ZnPP or tin protoporphyrin IX (SnPP) followed by hemin treatment increased the subG1-population, apoptosis and cytotoxicity in different CRC cell lines as well as non-cancerous HCEC [21,172,174,183,192]. In contrast to these findings, there are also studies that report apoptosis induction by high HO-1 expression upon CoPP exposure. In COLO205, HCT15, and LOVO cells the CoPP-induced HO-1 expression was related to higher subG1-population and higher expression levels of the apoptotic markers cleaved caspase-3 and cleaved PARP1. However, there was no increase in apoptosis in HT-29 cells with CoPP-induced high HO-1 expression [193]. Andres and colleagues revealed a significantly higher ratio of cells in the G0/G1-phase as well as higher apoptotic ratios after hemin-triggered HO-1 induction in HCT116 cells, while these effects depended on the p53 status [151]. HCT116 p53^−/−^ cells showed a significantly higher ratio of cells in the G2/M-phase and cell viability after HO-1 induction by hemin. Thus, p53 and/or its downstream targets like p21 seem to be involved and responsible for the HO-1 pro-apoptotic and anti-apoptotic effects. In human MCF-7 breast cancer cells, higher expression of p53 and p21 by hemin-induced HO-1 and its product ferrous iron was confirmed. It was shown that the iron chelators desferrioxamine and 1,10-phenanthroline counteract the p53 induction [63].

### 8.4. The Contribution of HO-1 to Ferroptosis

Ferroptosis is as an iron-dependent type of programmed, caspase-independent cell death, which is morphologically, biochemically, and genetically distinct from apoptosis, autophagy-dependent cell death, and necroptosis [194,195]. Interestingly, neurodegenerative diseases like Alzheimer and Parkinson are associated with higher cellular iron levels and ferroptosis, which could be a relevant origin of these diseases [196,197]. On the morphological level, ferroptosis is characterized by a shrinkage of mitochondria and a reduced number of mitochondrial cristae [198]. Ferroptosis is triggered by a massive ROS-dependent lipid peroxidation in the plasma membrane and severe mitochondrial damage due to an impaired activity of glutathione peroxidase 4 (GPX4) and its redox cycle (Figure 7C) [194,199]. Biochemical markers of ferroptosis include a reduced expression of *GPX4* and *SLC7A11* (a subunit of the cysteine/glutamate Xc- Antiporter), an increased expression of *ACSL4* (long-chain-fatty-acid-CoA ligase 4) and *PTGS2* (prostaglandin-endoperoxide synthase 2; COX-2), depletion of cellular glutathione (GSH), decreased NADPH levels as well as massive lipid peroxidation [195,200]. Iron chelators (e.g., deferoxamin) and lipophilic antioxidants such as Trolox or ferrostatin-1 were shown to inhibit ferroptosis by blocking iron-mediated ROS formation [201,202]. Known inducers of ferroptosis are glutamate, drugs such as erastin and sorafenib, which inhibit the Xc-antiporter and prevent cysteine import, as well as RSL3, which acts as a GPX4 inhibitor [195]. Both the p53 and Nrf2 pathway are also implicated in ferroptosis [195].

Heme degradation by HO-1 generates BV, CO, and ferrous iron. It is thus hypothesized that an increased HO-1 activity could result in ferrous iron accumulation, which may promote ferroptosis via the mechanism described above. Nevertheless, free ferrous iron (Fe^2+^) is oxidized to ferric iron (Fe^3+^) and stored by ferritin or directly exported out of the cell by FPN-1, which was shown to be an effective protection mechanism against ferroptosis [115,116]. HO-1 knockdown in non-malignant human colonocytes did not affect the expression of ferritin, but heme as the HO-1 substrate induces the expression of ferritin, which was shown in normal human colonocytes and human CRC cells [21,203]. Heme-treated Caco-2 CRC cells showed higher ferrous iron levels, followed by an increase in lipid peroxidation and signs of ferroptotic cell death [203]. A heme-induced increase in ferroptosis by additional treatment with erastin was also seen in human fibrosarcoma cells [204]. These studies suggest that HO-1 induction by heme and subsequent ferritin upregulation is not sufficient to protect the cells from iron accumulation and to inhibit ferroptosis. Interestingly, the incubation with high doses of ferrous and ferric iron (FeCl_3_ and FeSO_4_) did not or only marginally reduce the viability in human CRC cell lines and normal human colonocytes, while heme showed dose-dependent cytotoxicity preferentially in normal human colonocytes [21]. Thus, heme possess a higher cytotoxic potential than ferrous or ferric iron, but the underlying mechanism of heme-induced cell death in human colonocytes is still unclear and currently under investigation. 

Even if the HO-1-mediated heme degradation is a detoxification step, the role of HO-1 in iron deposition and ferroptosis in colon epithelial cells is still unknown. Nevertheless, ferroptosis may be able to regulate HO-1 and ferritin expression [205]. In human hepatic cancer cells the induction of ferroptosis by erastin led to significantly higher levels of the p62-KEAP1 complex [205]. The interaction of p62 with its Nrf2 binding site is known to inactivate KEAP1 [206,207] and thus allows Nrf2 to translocate into the nucleus and induce target genes (e.g., *HMOX1*, *Fth1*, and NAD(P)H quinone oxidoreductase 1 (*NQO1*)) [205,207,208]. A detailed overview of Nrf2 and BACH1 target genes in ferroptosis regulation has recently been published [209].

In addition to the direct interaction between HO-1 and ferroptosis, there are some ROS-protective mechanisms, which also influence this mutual interaction. GSH is an effective cellular antioxidant, which can reduce ROS, while at the same time being oxidized to glutathione disulfide (GSSG; Figure 7C) [210]. GPX4 catalyzes the reduction of hydrogen peroxide to water using GSH as a cofactor, whereby GSH is constantly regenerated by the NADPH-dependent glutathione reductase [211,212]. Therefore, both GSH and GPX4 are crucial factors inhibiting ferroptosis. On the one hand, it was shown that Nrf2 induced a significantly higher expression of GPX4 and glutathione synthase, while inhibition of Nrf2 by oxaliplatin reduced the GPX4 expression [172,209]. On the other hand, heme was reported to reduce GPX4 expression, which led to higher ROS levels and lipid peroxidation and further enhanced cellular sensitivity to ferroptosis [203,213]. Such a higher sensitivity was also described for heme treated lung and tubular epithelial cells [214,215]. In Caco-2 CRC cells, the induction of HO-1 by an herbal extract led to ferroptosis, which was characterized by higher ROS levels and lipid peroxidation without changes of the γ-glutamylcysteine synthetase (γ-GCS), a key enzyme in GSH synthesis [216]. To detail the role of HO-1 in ferroptosis of colon cells and tissue, future studies will be necessary by comparing the effects of heme iron versus non-heme iron with or without HO-1 regarding ROS, lipid peroxidation as well as cellular membrane damage.

### 8.5. Role of HO-1 in Tumor Initiation in Non-Colon Tissues

To sum up, HO-1 has a key function in the protection of colon cells and tissue against ROS, genotoxicity, and inflammation. The regulation of the cell cycle and apoptosis is affected by HO-1 expression, but further depends on the p53 status. Most of these preventive effects by HO-1 were also documented for other tissues. Transgenic mice with reduced HO-1 activity showed significantly higher levels of ROS and alanine transaminase in liver tissue, which are markers for liver injury [217]. The DNA-protective effects of high HO-1 expression was reported in vivo for the liver, kidney, and lung using HO-1-deleted mice as well as in isolated hepatic and peritoneal macrophages with HO-1 deletion [184,218,219]. In vitro, the induction of HO-1 reduced DNA damage as analyzed in monocytes and kidney cells [184,220]. While the impact of HO-1 on apoptosis in colon cells and tissue seems to be context-dependent, its role in other tissues is well established. In monocytes, papillary thyroid carcinoma cells and gastric cancer cells, HO-1 protected the cells from apoptosis and/or increased the G0/G1-population [220,221,222]. Moreover, the ratio of apoptotic cells in liver tissue of HO-1-depleted mice was significantly higher [217]. In turn, an increased HO-1 expression by CoPP led to lower apoptosis and higher cell viability in hepatic tissue of treated mice [223,224]. Similar results were seen in ovarian, breast, and pancreatic cancer cells, in which HO-1 inhibition significantly reduced the cell viability [174]. Even the survival of mice was elongated by high HO-1 expression and prevented the animals from chemically-induced inflammatory liver injuries [225]. The anti-inflammatory effect of HO-1 was shown in several studies using lung carcinoma cells or hepatic tissue of mice after HO-1 induction [218,224,225,226]. Here, HO-1 was able to reduce the expression of inflammation-promoting cytokines, which was also seen by incubation with CO and/or BV [224,225].

Furthermore, the role of HO-1 in liver tumor formation upon diethylnitrosamine (DEN) treatment was studied. DEN is a commonly used hepatic tumor initiator, which causes DNA ethylation adducts and promotes ROS formation after CYP-dependent metabolic activation [142,227,228,229]. Wild-type and transgenic mice with reduced HO-1 activity were treated with DEN followed by the characterization of liver tumors 45 weeks after initial DEN injection. The transgenic mice with reduced HO-1 activity showed a significantly higher relative liver weight, more tumors, as well as higher tumor area and diameter [217]. In another cancer model, liver cancer cells were injected in the liver lobe of mice, followed by siRNA mediated HO-1 knockdown. The treatment was continued for 10 days, while the HO-1 knockdown was induced in all tissue of the animals and not just in the injected liver cells. Interestingly, and in contrast to the first study, the inhibition of HO-1 reduced the formation of hepatic tumors [223]. Furthermore, the authors identified an increased tumor cell apoptosis due to HO-1 downregulation, which could be responsible for the significantly reduced tumor size. 

To sum up, HO-1 can prevent cells and tissue from damage, which could lead to tumor formation, while in established tumors and tumor cell lines the well-directed inhibition of HO-1 may increase apoptosis and reduce tumor size.

## 9. Role of HO-1 in Tumor Progression

As described above, HO-1 is involved in apoptosis induction and cell cycle regulation. Due to the balance between cell cycle arrest and proliferation, HO-1 could further affect cell proliferation and tumor growth (Figure 7D) [230]. When HO-1 was inhibited by ZnPP or SnPP in CRC cell lines (e.g., HCT-15, SW480 and Caco-2), their proliferation was significantly reduced, while HIF-1α level was reduced likewise [183,192,231]. HIF-1α, a subunit of the heterodimeric transcription factor HIF, was shown to be upregulated in more than 50% of CRC tissues samples, and is known to promote angiogenesis and tumor growth under hypoxic condition [231,232] Nevertheless, it is also closely related to cell proliferation and migration [233]. Both are essential properties, which are evolved during EMT, a key process in tumor progression and metastasis [150,162]. In HCT116 cells, hemin significantly reduced cell migration, while the HO-1 inhibitor ZnPP led to higher rates of migration as observed in the wound healing assay [151]. Contrary results were described in HT-29 and DLD-1 cells after HO-1 induction by glucose-related protein 78 (GRP78) knockdown [234]. The underlying mechanisms are still unclear, but it was shown that the GRP78 knockdown activates the Nrf2/HO-1 pathway. Moreover, cell migration, invasion, and the expression of EMT marker proteins such as vimentin were increased similar to HO-1 expression [234]. If these results were directly affected by GRP78 knockdown or by the increased HO-1 level is not clear. Another EMT-promoting effect of HO-1 was shown in ZnPP treated HCT-15 cells. The inhibition of HO-1 reduced tumor growth and angiogenesis in a xenograft mouse model, supporting the notion that HO-1 promotes EMT [231]. Moreover, the proliferation of injected C26 cells in the footpad of mice was slowed down in a dose-dependent manner by ZnPP treatment [174]. In conclusion, there are divergent results about the role of HO-1 during EMT of CRC cells. To clarify its role in EMT and tumor progression, future studies are required, including in vivo experiments, where the whole process of metastasis and tumor progression can be analyzed. Furthermore, the establishment and use of new approached methodologies (NAMs) should be promoted to replace and reduce animal testing [235]. 

In the case of non-colon tissue, the role of HO-1 in tumor progression is unclear and contrary effects were reported in the literature. In cancer cell lines derived from breast, ovary, and prostate cancer tissues, the induction of HO-1 reduced cell migration, invasion, proliferation as well as the expression of EMT marker proteins and the matrix metallopeptidase 9 (MMP-9) [93,236,237,238,239,240,241,242]. Moreover, the injection of HO-1 overexpressing breast cancer cells in mice led to reduced metastasis, tumor formation, and tumor growth [236]. MMP-9 is able to degrade collagen and gelatin, followed by the release of signal molecules. Therefore, the tight tissue structure becomes disintegrated and facilitates the access of tumor cells to blood vessels, while the signal molecule further promotes cell migration [243]. In lung cancer cells, both a positive and a negative effect of HO-1 on MMP expression was seen. Kang and colleagues showed a significant decrease in cell invasion and MMP-9 expression by HO-1 knockdown, whereas Tertil and coworkers reported a lower cell proliferation, migration and MMP induction by HO-1 overexpression [226,244,245]. The divergent role of HO-1 was also seen in liver cancer cells. On the one hand, HO-1 induction reduced inflammation-induced liver tumor formation in mice [218]. On the other hand, siRNA-mediated knockdown of HO-1 reduced cell proliferation, with lower levels of angiogenesis and tumor volume after injection of hepatic cancer cells in mice [223]. Finally, HO-1 was reported to promote EMT-related mechanisms in cervix cancer and bone osteosarcoma cells [246]. Induction of HO-1 increased cell proliferation, migration and invasion [246]. The results in kidney cancer cells were similar, revealing lower cell proliferation and anchorage independent growth in the colony formation assay after HO-1 inhibition [247]. The role of HO-1 in tumor progression of pancreatic and melanoma cancer cells were studied in vivo. Cancer cells with an overexpression of HO-1 were injected in mice, which resulted in higher levels of angiogenesis, metastasis, and tumor volume [248,249]. For the mice injected with the melanoma cancer cells a significantly lower survival rate was seen in the HO-1 overexpression group compared to the control group with normal HO-1 expression [249]. In conclusion, HO-1 can promote or prevent tumor progression-related mechanisms, which depends on the origin tissue. Breast and prostate cancer cells were inhibited by HO-1, while its function in liver and lung cells is controversially discussed similar to the CRC studies. In conclusion, data on the role of HO-1 in colorectal tumor progression are limited, while tumor-promoting effects like increased tumor growth and angiogenesis as well as inhibiting effects, e.g., a reduced cell migration by HO-1 are documented. Similar contrary results of the contribution of HO-1 in tumor progression were reported for breast, lung, and hepatocellular tissue, highlighting the need for further in vitro and in vivo studies, e.g., with cell-specific HO-1 knockout or HO-1 overexpression. 

## 10. Conclusions

HO-1 is the main enzyme responsible for the cellular heme degradation, thereby forming ferrous iron, CO, and BV. In contrast to its isoform HO-2, which is constitutively expressed in neurons and other tissues, HO-1 occurs in all mammalian tissues and is strongly inducible by Nrf2 activation upon oxidative stress and heme-dependent inactivation of its repressor BACH1. In addition to its cytoprotective role towards toxic free heme and ROS, HO-1 contributes to cellular homeostasis, as shown in different knockout mouse models and human case reports with sporadic HO-1 loss of function mutations. Those patients suffer from chronic inflammation and elevated ROS levels, which can drive tumorigenesis. Several studies provided evidence that the heme degradation product CO can promote DNA repair and exerts anti-inflammatory effects, while BV acts as an antioxidant and may thus prevent ROS-induced genotoxicity. With regard to apoptosis and inflammatory response, the role of HO-1 in colon (cancer) tissue and cells is inconsistent. This also holds true for tumor progression-related mechanisms. On the one hand, HO-1 induction was reported to block cell migration. On the other hand, HO-1 inhibition was found to reduce tumor growth and angiogenesis.

To sum up, the role of HO-1 in colorectal carcinogenesis is insufficiently understood and needs to be analyzed in more detail, e.g., by direct comparison of cancer-related effects in HO-1 overexpressing and HO-1 knockout cell lines as well as appropriate mouse models with tissue-specific HO-1 deletion or overexpression.

## Data Availability

Not applicable.

## Abbreviations

γ-GCS	γ-glutamylcysteine synthetase
ACSL4	Long-chain-fatty-acid-CoA ligase 4
AKI	Acute kidney injury
AMPK	AMP-activated protein kinase
AP-1	Activator protein-1
AP-2	Activator protein-2
ARE	Antioxidant response element
BACH1	BTB and CNC homology 1
BK	Calcium-sensitive potassium
BR	Bilirubin
BV	Biliverdin-IXα
BVR	Biliverdin reductase
bZIP	Basic-region leucine zipper
Caco-2	Human epithelial colorectal adenocarcinoma cells
CD	Crohn’s disease
CK2	Casein kinase 2
CNC	Cap’n’collar
CoPP	Cobalt protoporphyrin IX
CRC	Colorectal cancer
Cul3	Cullin 3
DDS	Dextran sodium sulphate
DEN	Diethylnitrosamine
ECE-1	Endothelin-converting enzyme-1
Egr-1	Early growth response protein 1
EMT	Epithelial-mesenchymal transition
ER	Endoplasmatic reticulum
ERK	Extracellular signal-regulated kinase
FPN	Ferroportin
FtH	Ferritin heavy chain
FtL	Ferritin light chain
GPX4	Glutathione peroxidase 4
GRP78	Glucose-related protein 78
GSH	Glutathione
GSSG	Glutathione disulfide
HCEC	Human colonic epithelial cells
HIF-1α	Hypoxia-inducible factor-1
HMEC-1	Human microvascular endothelial cell line
HO-1	Heme oxygenase-1
HO-2	Heme oxygenase-2
HO-3	Heme oxygenase-3
HRMs	Heme-regulatory motifs
HUVEC	Human umbilical vein endothelial cells
IBD	Inflammatory bowel disease
ICAM-1	Intercellular adhesion molecule 1
ICH	Intracerebral haemorrhage
IFN-γ	Interferon-γ
IL-1β	Interleukin-1β
IL-6	Interleukin 6
IR	Ischemia-reperfusion
IRI	Ischemia-reperfusion injury
JNK	C-Jun N-terminal kinase
KEAP-1	Kelch-like ECH-associated protein 1
KRAS	Kirsten-ras
LPS	Lipopolysaccharide
MAPK	Mitogen-activated protein kinase
MIP-1β	Macrophage inflammatory protein-1β
miRs	MicroRNAs
MMP-9	Matrix metallopeptidase 9
MPO	Myeloperoxidase
MSCs	Mesenchymal stem cells
NAMs	New approached methodologies
NF-κB	Nuclear factor-κB
NQO1	NAD(P)H quinone oxidoreductase 1
Nrf2	Nuclear factor erythroid 2-related factor 2
PI3K	Phasphatidylinositol 3-kinase
PKC	Protein kinase C
PPARγ	Peroxisome proliferator-activated receptor-γ
PTGS2	Prostaglandin-endoperoxide synthase 2
ROS	Reactive oxygen species
sMAF	Small musculoaponeurotic fibrosarcoma
SnPP	Tin protoporphyrin IX
SPF	Specific pathogen-free
STAT	Signal Transducer and Activator of Transcription
TBARS	Thiobarbituric acid reactive substances
TCR -α	T cell receptor-alpha
TJ	Tight junction
TLR	Toll-like receptor
TNFα	Tumor necrosis factor-α
TPP	Tristetraprolin
UC	Ulcerative colitis
VCAM-1	Vascular cell adhesion protein 1
ZnPP	Zinc protoporphyrine IX
